# Clinical PDT dose dosimetry for pleural Photofrin-mediated photodynamic therapy

**DOI:** 10.1117/1.JBO.29.1.018001

**Published:** 2024-01-13

**Authors:** Hongjing Sun, Yihong Ong, Weibing Yang, Dennis Sourvanos, Andreea Dimofte, Theresa M. Busch, Sunil Singhal, Keith A. Cengel, Timothy C. Zhu

**Affiliations:** a University of Pennsylvania, Department of Radiation Oncology, Philadelphia, Pennsylvania, United States; b University of Pennsylvania, Department of Bioengineering, Philadelphia, Pennsylvania, United States; c University of Pennsylvania, School of Dental Medicine, Department of Periodontics, Philadelphia, Pennsylvania, United States; d University of Pennsylvania, Schools of Engineering and Dental Medicine, Center for Innovation and Precision Dentistry, Philadelphia, Pennsylvania, United States; e University of Pennsylvania, Department of Surgery, Philadelphia, Pennsylvania, United States

**Keywords:** photodynamic therapy, photodynamic therapy dose, Photofrin, pleural photodynamic therapy, IR navigation system

## Abstract

**Significance:**

Photodynamic therapy (PDT) is an established cancer treatment utilizing light-activated photosensitizers (PS). Effective treatment hinges on the PDT dose-dependent on PS concentration and light fluence-delivered over time. We introduce an innovative eight-channel PDT dose dosimetry system capable of concurrently measuring light fluence and PS concentration during treatment.

**Aim:**

We aim to develop and evaluate an eight-channel PDT dose dosimetry system for simultaneous measurement of light fluence and PS concentration. By addressing uncertainties due to tissue variations, the system enhances accurate PDT dosimetry for improved treatment outcomes.

**Approach:**

The study positions eight isotropic detectors strategically within the pleural cavity before PDT. These detectors are linked to bifurcated fibers, distributing signals to both a photodiode and a spectrometer. Calibration techniques are applied to counter tissue-related variations and improve measurement accuracy. The fluorescence signal is normalized using the measured light fluence, compensating for variations in tissue properties. Measurements were taken in 78 sites in the pleural cavities of 20 patients.

**Results:**

Observations reveal minimal Photofrin concentration variation during PDT at each site, juxtaposed with significant intra- and inter-patient heterogeneities. Across 78 treated sites in 20 patients, the average Photofrin concentration for all 78 sites is 4.98  μM, with a median concentration of 4.47  μM. The average PDT dose for all 78 sites is $493.17\;\mu \mathrm{MJ}/{\mathrm{cm}}^{2}$, with a median dose of $442.79\;\mu \mathrm{MJ}/{\mathrm{cm}}^{2}$. A significant variation in PDT doses is observed, with a maximum difference of 3.1 times among all sites within one patient and a maximum difference of 9.8 times across all patients.

**Conclusions:**

The introduced eight-channel PDT dose dosimetry system serves as a valuable real-time monitoring tool for light fluence and PS concentration during PDT. Its ability to mitigate uncertainties arising from tissue properties enhances dosimetry accuracy, thus optimizing treatment outcomes and bolstering the effectiveness of PDT in cancer therapy.

## 1 Introduction

Photodynamic therapy (PDT) is an evolving treatment modality that has gained approval from the U.S. Food and Drug Administration for the treatment of various cancers, including microinvasive lung cancer, obstructing esophageal cancer, and non-small cell lung cancer. It is also utilized for premalignant conditions, such as Barrett’s esophagus, and non-oncologic conditions, such as age-related macular degeneration.^1^^–^^4^ PDT offers valuable advantages, such as minimal invasion, low systemic toxicity, versatility, and repeatability. However, the optimal clinical outcomes of PDT have been hindered by the lack of a reliable dosimetry metric that accurately quantifies the effective treatment dose, which is essential for predicting and guiding PDT.^5^ The challenges in achieving accurate dosimetry for PDT stem from its dynamic and complex nature, involving intricate interactions between light, the photosensitizer (PS), and tissue oxygen (O23).^6^ In a typical PDT process, the PS is excited by specific wavelengths of treatment light, causing it to transition from its ground state to the excited singlet state. Subsequently, the PS undergoes intersystem crossing, leading to its transition to the triplet state. Type II PDT, the most clinically relevant process, occurs when the triplet state transfers energy to ground-state oxygen, O23, generating singlet oxygen, O21. This singlet oxygen induces photodamage in the photosensitized area.^7^^–^^11^

Conventional PDT dosimetry relies on a prescribed administered drug dose and the total light fluence, which represents the energy per unit area delivered by the end of treatment. Although these basic dosimetry metrics can be accurately and easily monitored, they often prove insufficient due to significant inter- and intra-patient variability in PS localization and the tumor microenvironment.^12^^–^^14^ Thus, the improvement of dosimetry methods is crucial for advancing the application of PDT. The PDT dose, defined as the temporal integral of the product of *in vivo* PS concentration and light fluence in the target tissue, has been demonstrated in several preclinical studies as a more effective dosimetric quantity than light fluence or PS concentration alone, particularly under well-oxygenated conditions.^5^^,^^15^^–^^17^

Our research group has developed a dosimeter that enables the simultaneous measurement of light fluence rate and PS fluorescence during treatment. This dosimeter has been successfully employed in clinical measurements as part of an ongoing phase II/III clinical trial of Photofrin-mediated PDT for pleural mesothelioma.^18^^–^^20^ The measured PS fluorescence at the tissue surface allows for the calculation of PS concentration. Together with the measured light fluence, this information facilitates the determination of the PDT dose delivered to the superficial tissue of the pleural cavity. We have observed spatial heterogeneities in the delivered PDT dose on the cavity surface due to complex pharmacokinetics and tissue conditions.^14^ Consequently, it is crucial for the dosimetry system to cover the entire cavity surface rather than focusing on a single isolated point. To address this, the newly developed dosimetry system features eight dual-function channels, enabling the monitoring of eight different locations. The primary aim of this study is to provide updated information on PDT dose variations within the context of our pleural PDT clinical trial. Notably, the study expands its scope by increasing the number of patients from 8 to 20 and the inclusion of sites from 22 to 78.^14^^,^^21^ Furthermore, our research involves a thorough and detailed examination of the obtained data. This includes a comprehensive reanalysis of fluorescence data to derive PS uptake, a thorough reevaluation of tissue optical properties through Diffuse Reflectance Spectroscopy (DRS) data, and a complete reassessment of optical property correction factors for determining PS uptake in both patients and phantoms. Through these efforts, we address previously existing gaps in the literature, providing a more comprehensive and updated understanding of the subject matter. In addition, we aim to incorporate an optical infrared (IR) navigation system to provide comprehensive dosimetry guidance throughout the pleural PDT procedure. Although existing real-time dosimetry methods primarily focus on *in-vivo* light fluence measurements, the use of innovative instruments makes real-time PDT dose evaluation feasible. This study provides a summary of the current state of the art in multichannel PDT dose measurements for clinical applications and explores the various potentials of this versatile dosimetry system.

## 2 Materials and Methods

### 2.1 Clinical Trial and Concurrent Measurements

**Fig. 1:**
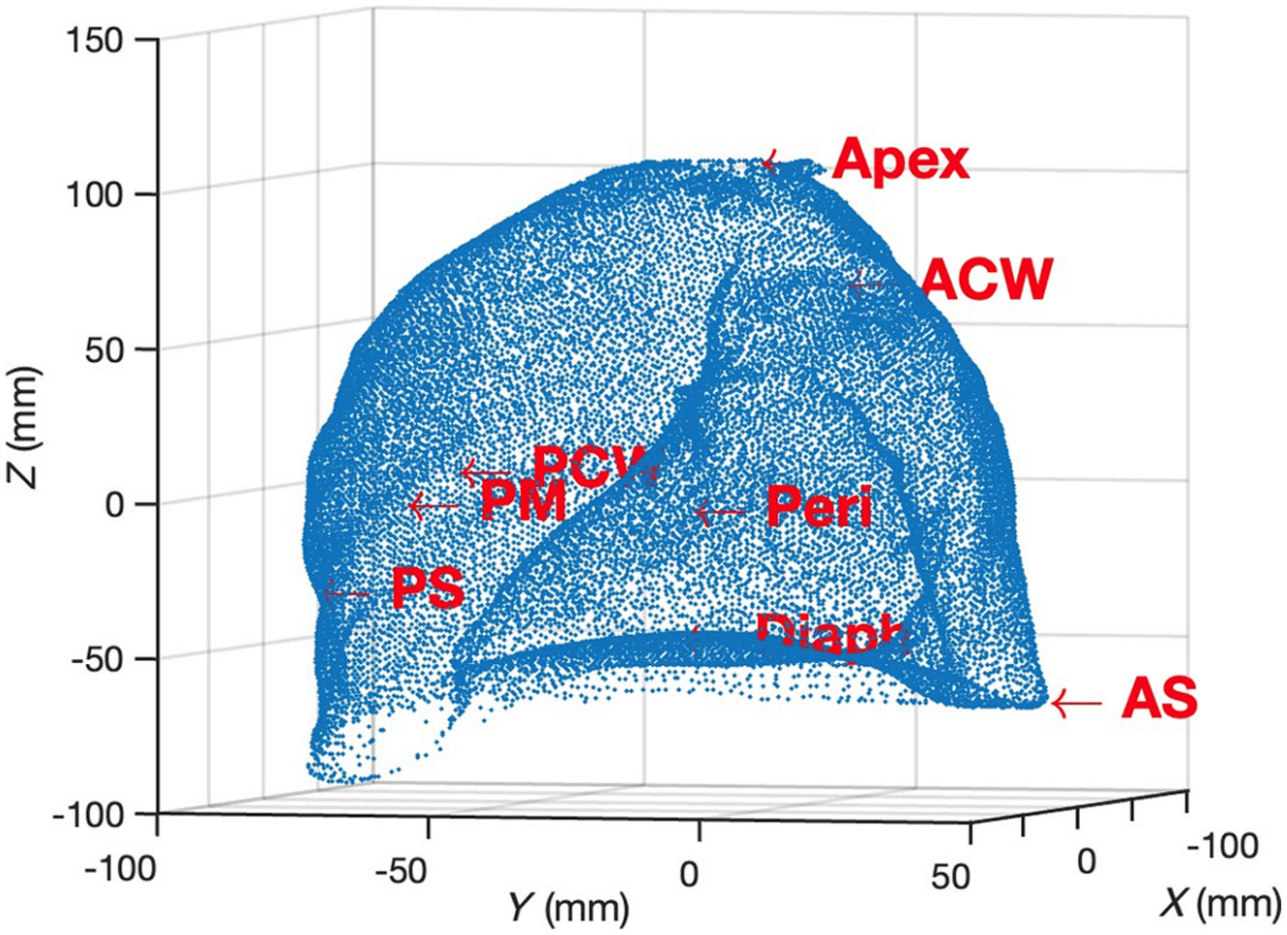
Point cloud data of a reconstructed pleural cavity, with isotropic locations marked, were generated using our scanning system.

Patients with pleural mesothelioma were enrolled in a phase II/III randomized clinical trial at the Hospital of the University of Pennsylvania after providing informed consent. A subset of patients underwent lung-sparing surgical resection combined with interoperative Photofrin-mediated PDT, and the remaining patients received surgery alone. Further details regarding the PDT technique and treatment protocol can be found elsewhere.^22^ For patients receiving PDT, Photofrin (Pinnacle Biologics, Chicago, Illinois) was administered intravenously at a dose of 2 mg per kg of body weight, 24 h prior to PDT. PDT was performed immediately following surgery using a KTP-pumped dye laser (model 630 XP, Laserscope, Inc., San Jose, California) with 632 nm light. The prescribed total light fluence was set at $60\;J/{\mathrm{cm}}^{2}$. The instantaneous light fluence rate was measured using eight isotropic detectors (Medlight SA, Ecublens, Switzerland) sutured at the apex, posterior chest wall (PCW), anterior chest wall (ACW), posterior sulcus (PS), anterior sulcus (AS), posterior mediastinum (PM), pericardium (Peri), and diaphragm (Diaph)^23^ on the pleural cavity surface (see Fig. 1). The cumulative light fluence was simultaneously calculated until the prescribed light dose was achieved at all locations. In addition to the light fluence rate, the Photofrin fluorescence excited by the treatment light was monitored using the same eight isotropic detectors. The light was delivered via a bare optical fiber embedded in a modified endotracheal tube. To facilitate light scattering, the treatment tube and pleural cavity were filled with 0.1% Intralipid. The physician sequentially moved the light source along the inner surface of the cavity during PDT until the prescribed light dose was reached.^24^

Accurately quantifying fluorescence emission *in vivo* is challenging due to the varying optical properties of the surrounding tissue. To mitigate this effect, diffuse reflectance measurements were obtained on the inner surface of the pleural cavity before and after PDT treatment for optical property correction. A custom-made fiber optic-based contact probe (FiberOptic Systems, Inc., Simi Valley, California) was employed, consisting of one source fiber connected to an air-cooled quartz-tungsten-halogen lamp (Avalight HAL-S, Avantes, Inc., Louisville, Colorado) and nine detection fibers spaced at distances ranging from 1.4 to 8.7 mm from the source fiber. The detection fibers captured the diffuse reflected light, which was then directed to a spectrograph and recorded on a charge-coupled device (CCD)-based camera sensor (InSpectrum 150, Roper Scientific, Princeton, New Jersey) for radially resolved diffuse reflectance measurement. Background signals were concurrently measured for subsequent subtraction. The acquired spectra were fitted using a nonlinear fitting algorithm implemented in the MATLAB programming environment (The MathWorks, Natick, Massachusetts) to extract the tissue optical properties at each measured location. Further information regarding the probe design and fitting algorithm can be found elsewhere.^21^^,^^25^

### 2.2 PDT Dose Dosimeter Instrumentation

**Fig. 2:**
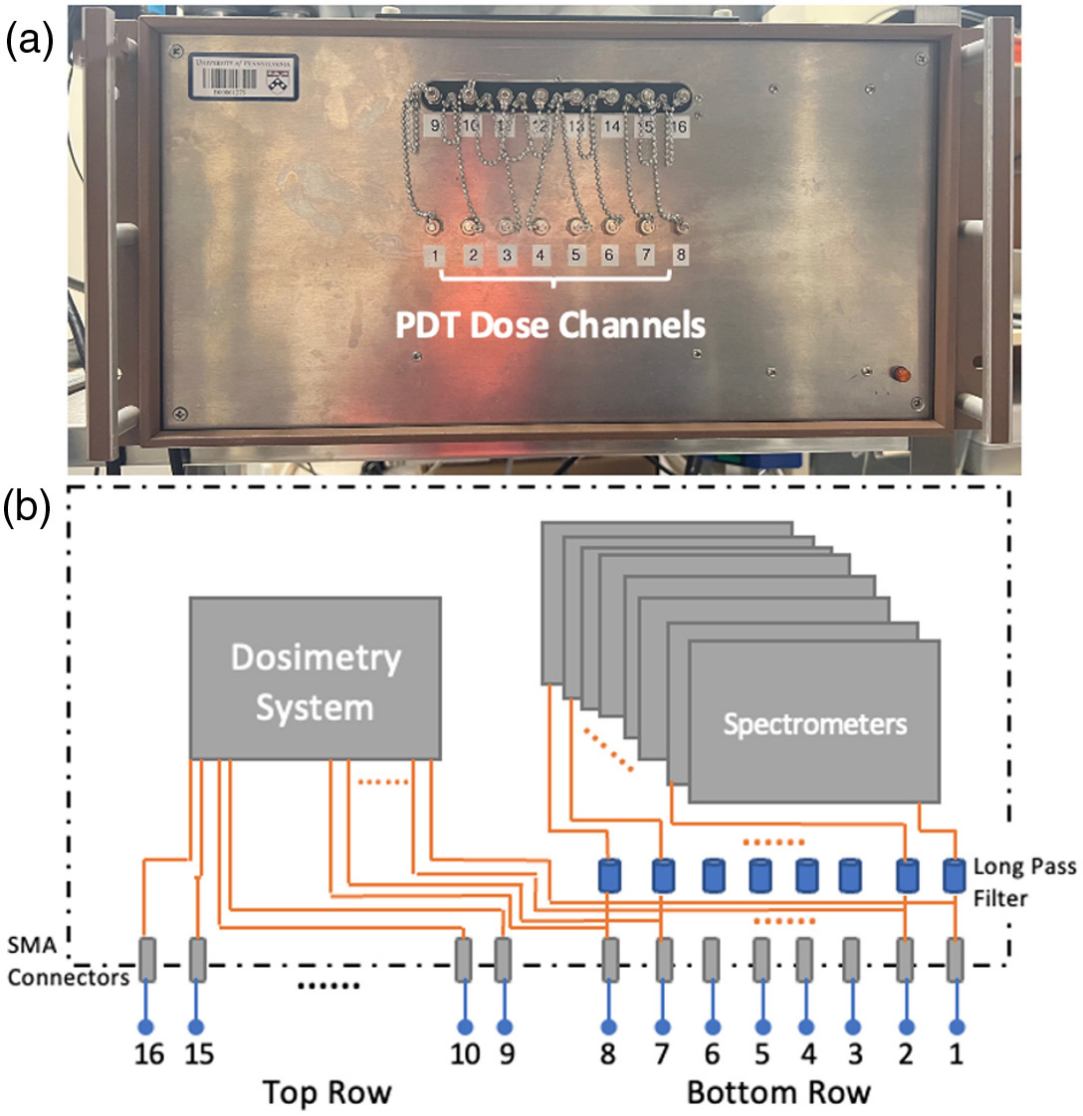
(a) The front view of the 8-channel PDT dose dosimetry instrument and (b) the schematic diagram of the system setup. The bifurcated fibers are connected to channels 1–8 internally, enabling the simultaneous measurement of light fluence and PS uptake. A long-pass filter is employed for each channel, leading to a slight variance in the peak wavelength of the measured spectrum. To ensure independent functionality, distinct long-pass filters are employed for each channel.

The comprehensive multichannel dosimetry system incorporated the use of eight isotropic detectors that were sutured onto the interior pleural surface. These detectors were connected to individual channels within the dosimetry system. Figure 2 provides a front view of the multichannel dosimetry system and a schematic diagram of its internal setup. The system enclosure contained a total of 16 channels [Fig. 2(a)]. The internal structure of the equipment is shown in Fig. 2(b). The bottom row was comprised of channels 1 to 8, with each channel split into two fibers through 1-to-2 bifurcated fibers (Ocean Optics, Dunedin, Florida) internally inside the box. The bifurcated fibers of each channel were connected to the photodiodes and one spectrometer (Exemplar, B&W Tek, Inc., Newark, Delaware) for the simultaneous monitoring of the fluence rate of the treatment light and Photofrin fluorescence. To block the treatment light, a long-pass filter was installed for each channel in conjunction with the spectrometer. Due to space constraints, the remaining channels (channels 9 to 16) do not have the bifurcations, so they cannot measure the PS uptake and were solely connected to the dosimetry system. The PDT dose dosimetry instrument always had 16 channels for light dosimetry and was incrementally expanded to 8 channels to measure the PS uptake and light fluence rate simultaneously.

The spectrometers were utilized for measuring the Photofrin fluorescence, and the photodiodes served as monitors for the fluence rate of the treatment light. Recently, the dual-function channels were expanded from four to eight, allowing for a more comprehensive measurement of the entire cavity. The spectrometer had a wavelength range of 200 to 1050 nm and a resolution of 0.42 nm using a diode array with dimensions of 2048×1 elements and an element size of 14  μm×200  μm. The achieved spectral resolution was 0.47 nm. The isotropic detector directly measured the light fluence rate at the surface of the cavity. However, it should be noted that this measurement might differ from the intra-tissue light fluence rate due to variations in tissue optical properties. To ensure the accurate measurement of the transmitted fluorescence (signal after 633 nm), long pass filters (Semrock, Inc., Rochester, New York) were employed to block the treatment light. No filtration was required for the treatment-light signal prior to reaching the photodiodes.^26^ In this study, all eight isotropic detectors were utilized and connected to channels 1 to 8 to simultaneously measure the fluence rate of the treatment light and Photofrin fluorescence throughout the pleural cavity.

### 2.3 Spectroscopy

**Fig. 3:**
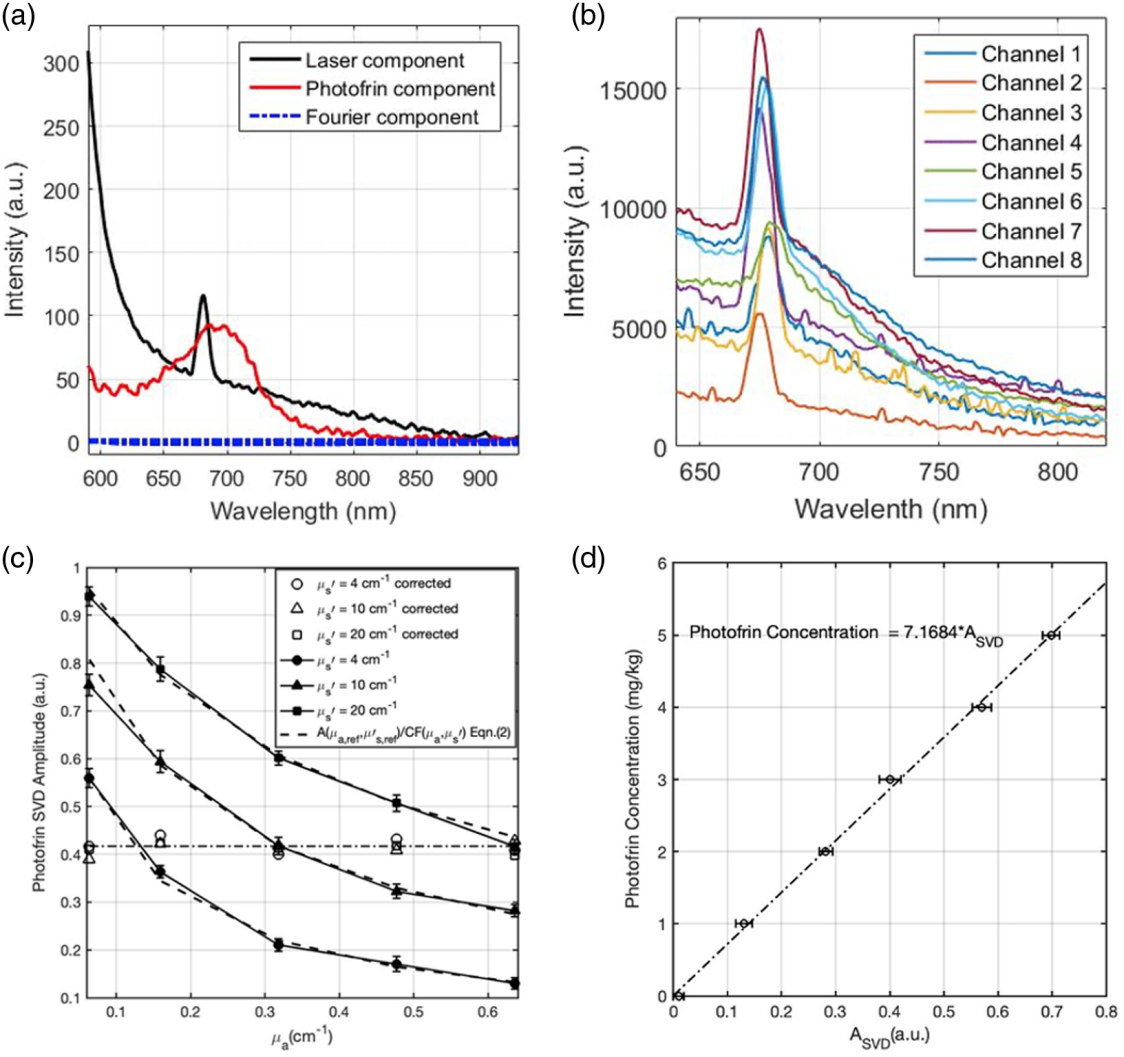
(a) The basis spectrum consists of a laser component, a Photofrin fluorescence component, and a Fourier component; (b) example of measured raw fluorescence spectra from 8 channels of patient #53. The peaks at 675 nm arise from fluorescence of the isotropic detector. Slight shifts in the peak wavelength occur as a result of using different long-pass filters for each channel. (c) Fluorescence SVD amplitudes for Photofrin in tissue-simulating phantom experiments with different optical properties ($\mu_{a}=0.06-0.7\;{\mathrm{cm}}^{-1}$ and $\mu_{s}^{\prime }=4$ to $20\;{\mathrm{cm}}^{-1}$) with a constant Photofrin concentration of $3\;\mathrm{mg}\;{\mathrm{kg}}^{-1}$, measured in phantoms using 8-channel PDT dosimeter (solid lines) and $A(\mu_{a},\mu_{s}^{\prime })$ fits using Eq. (2) (dashed lines). The correction factor (CF) and the corrected Photofrin SVD amplitudes were obtained using Eqs. (1) and (2). (d) Photofrin concentration calibration curve.

Fluorescence spectra were collected for eight strategic locations within the pleural cavity wall, as described in Sec. 2.1 and shown in Fig. 1, using eight single-channel CCD spectrometers. To evaluate the concentration of Photofrin, the raw fluorescence spectra obtained during PDT were corrected for the spectral response of each spectrometer. Subsequently, a single value decomposition (SVD) fitting algorithm was applied to analyze the spectra using a basis spectrum [Fig. 3(a)].^27^ The basis spectrum consisted of a laser component and a Photofrin fluorescence component, which were established through extensive phantom studies. The laser component served as a reference for the excitation light intensity. Background spectra were measured and subtracted. In the SVD algorithm, a 21-term Fourier series was incorporated to account for any unknown spectroscopic components, such as ambient room light. However, its weight in the fitting routine was lower than that of the measured fluorescence emission components.

Figure 3(b) presents an example of representative raw fluorescence spectra measured from the eight channels of patient #53. Throughout the treatment, each channel captured hundreds of fluorescence spectra, which were individually fitted using the SVD algorithm. The peaks observed around 675 nm corresponded to the fluorescence signal of Photofrin detected by the isotropic detectors. Slight shifts in the peak wavelengths were observed across different channels, attributed to the distinct long-pass filter characteristics of each channel. To accommodate this, during SVD fitting, adjustments were made to the basis spectrum to align with the shifted peaks for different channels. By fitting the raw measured spectra with the basic components, unitless SVD amplitudes were obtained. The laser components enabled the elimination of variations in the excitation light, allowing for the quantification of the local Photofrin concentration. A linear relationship was found between the SVD amplitudes and the local Photofrin concentration, enabling the quantification by multiplying with a constant. The determination of this constant was demonstrated in a previous study.^26^ The SVD fitting method utilized a large number of fluorescence spectra measured throughout the treatment at each location, leading to a significant reduction in uncertainties. The SVD fitting results were later adjusted to accommodate distortions arising from variations in optical properties, as elaborated in detail in Sec. 2.4.

### 2.4 Optical Property Correction

Accurately quantifying the Photofrin concentration using raw fluorescence spectra captured by the spectrometer has proven challenging due to the complexity of the surrounding tissues.^28^^–^^31^ To account for the influence of different optical properties on the detected fluorescence signal, a correction factor based on absorption coefficient ($\mu_{a}$) and scattering coefficient ($\mu_{s}^{\prime }$) was introduced.^24^^,^^32^ This correction factor, CF, was determined using a series of tissue-simulating phantoms with varying optical properties ($\mu_{a}=0.06$ to $0.7\;{\mathrm{cm}}^{-1}$ and $\mu_{s}^{\prime }=4$ to $20\;{\mathrm{cm}}^{-1}$) while keeping the Photofrin concentration constant at $3\;\mathrm{mg}\;{\mathrm{kg}}^{-1}$. Intralipid (Fresenius Kabi, Germany) was used as a light scatterer, and ink (Parker^®^ Quink^®^) served as the light absorber in the phantoms. The values of $\mu_{a}$ and $\mu_{s}^{\prime }$ represent the absorption and reduced scattering coefficients, respectively, at the emission wavelength of 630 nm, corresponding to the Photofrin fluorescence. The fluorescence signals from these phantoms were measured using the 8-channel PDT dosimeter. The raw fluorescence spectra obtained were then fitted using the SVD fitting algorithm described in the previous section, yielding an SVD amplitude ($A_{\mathrm{SVD}}$) for each phantom, as shown in Fig. 3(c). An empirical formula fitting the experimental data was employed to calculate the optical property correction factor, *CF*, from a given tissue optical properties $\left(\mu_{a},\mu_{s}^{\prime }\right)$ to that of the reference tissue optical properties ($\mu_{a,\mathrm{ref}}=0.32\;{\mathrm{cm}}^{-1}$ and $\mu_{s,\mathrm{ref}}^{\prime }=10\;{\mathrm{cm}}^{-1}$):

$$\mathrm{CF}=C_{1}(\mu_{a}^{b_{1}}\mu_{s}^{\prime b_{2}}+C_{2}),$$ (1)

where parameters $C_{1}=15.30\pm 0.52$, $C_{2}=0.023\pm 0.013$, $b_{1}=0.85\pm 0.15$, and $b_{2}=-0.95\pm 0.09$, were determined from fitting by Origin Pro 2023b (OriginLab Corp., Northampton, Massachusetts). The SVD amplitude obtained from the fluoresce spectrum is corrected by the calculated CF:

$$A_{\mathrm{corr}}(\mu_{a,\mathrm{ref}},\mu_{s,\mathrm{ref}}^{\prime })=CF(\mu_{a},\mu_{s}^{\prime })\cdot A(\mu_{a},\mu_{s}^{\prime }).$$ (2)

Monte Carlo calculations were conducted to determine the fitting function and its parameters.^26^^,^^33^ A different set of tissue-simulating phantoms, featuring varied Photofrin concentrations ranging from 0 to 5  mg/kg, was employed to create a calibration curve for the $A_{\mathrm{cor}}$ and the absolute Photofrin concentrations. The resulting calibration curve is shown in Fig. 3(d). This calibration curve enables the determination of absolute Photofrin concentrations by utilizing the optical property correction factor ($A_{\mathrm{cor}}$).

### 2.5 Real-Time Scanning and Navigation System

**Fig. 4:**
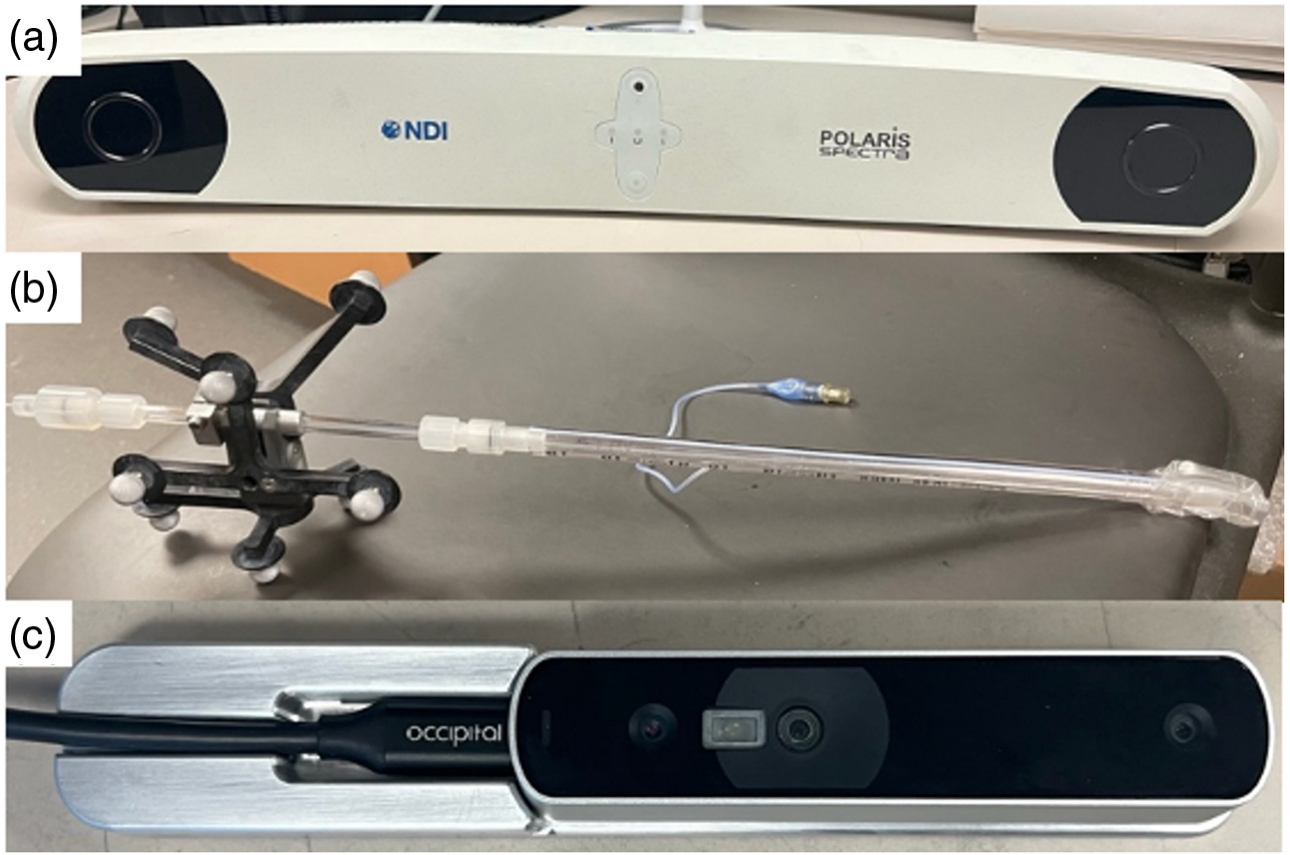
Instruments consist of the newly developed real-time navigation system. The optical infrared (IR) navigation system is made up of (a) an IR camera and (b) a treatment wand with a laser fiber inserted. The novel scanning system is made up of (c) a 3D scanning device for capturing the pleural surface.

To enhance the prediction and efficacy of PDT, our research group has developed a comprehensive real-time scanning and navigation system, in addition to the light dosimetry system. This system consists of a commercial optical infrared (IR) camera (Polaris, NDI, Waterloo, Canada), trackable wands, and a 3D scanner, as shown in Fig. 4. The hand-held 3D scanner (Structure Core, Occipital, Boulder, Colorado) is utilized for rapid and precise capture and reconstruction of the pleural cavity, enabling the identification of the target surface for real-time calculation of light fluence distribution during PDT.^34^ The IR navigation system comprises stereo cameras, a modulated laser source (wavelength 850 nm), and a treatment wand equipped with the PDT light source and nine passive markers. The cameras track the position of the light source in real time by detecting the light reflected from the markers on the wand at a rate of 20 to 60 Hz. This navigation system has been successfully employed in clinical trials for both HPPH- and Photofrin-mediated pleural PDT. The collected data from these trials have been utilized for post-treatment analysis, providing valuable insights into the treatment process and facilitating outcome prediction.^35^ By incorporating this innovative scanning and navigation system alongside the dosimetry system, we can provide more comprehensive real-time guidance for PDT, leading to improved treatment efficiency.

To provide real-time feedback on the light fluence distribution across the entire inner surface of the cavity, a newly developed graphical user interface (GUI) was implemented.^36^^,^^37^ This GUI enables the visualization of the light fluence distribution in real time. Simultaneously, the light dosimetry system was utilized to measure the light fluence for verification purposes. It should be noted that the light fluence at each point of interest on the inner surface of the cavity is the sum of both the primary component and the scattered component of the treatment light.^22^ The GUI provides a comprehensive and real-time representation of the light distribution, allowing for enhanced monitoring and optimization of the PDT procedure. The primary component of the light fluence rate (φ) is calculated as

$$\phi =\frac{S}{4\pi r^{2}},$$ (3)

where S is the source power (mW) and r is the distance (mm) between the point light source and the cavity surface. For more accurate calculation, scatter light fluence rate is included; it is calculated as

$$\phi =\frac{S}{4\pi r^{2}}+b,$$ (4)

where b is an empirical constant.^23^^,^^37^ The ultimate goal is to integrate this innovative system with the newly developed 8-channel dosimetry system, enabling the provision of real-time PDT dose information for the pleural cavity during PDT.

## 3 Results and Discussion

### 3.1 Tissue Optical Properties and Correction Factors

In this study, *in vivo* diffuse reflectance measurements were conducted at 52 sites within the pleural cavities of 11 patients. Initially, the PDT dose dosimetry system had four channels capable of simultaneously measuring light fluence rate and fluorescence. However, for the latest two patients, the system was expanded to eight channels, covering all eight predetermined locations within the pleural cavity for each patient. In this paper, the presented data for the four-channel system differ from previously reported results obtained using the same system. All raw data underwent thorough validation and were re-analyzed specifically for this study. Table 1 summarizes the tissue absorption and reduced scattering coefficients ($\mu_{a}$ and $\mu_{s}^{\prime }$) at the excitation wavelength of 630 nm for all measurement sites where diffuse reflectance spectra were obtained, along with the corresponding correction factors. Significant heterogeneity in both absorption and reduced scattering coefficients can be observed both within and among patients.

**Table 1:** Summary of absorption coefficients, scattering coefficients, and correction factors for 20 patients. The light fluence on the surface is the same at $60\;{\mathrm{Jcm}}^{-2}$ for all patients. Information about patients 07, 08, 12, 14, 16, 17, 18, and 20 can be found elsewhere and were reprocessed in this study.^26^

Patient	Site*	Optical properties		CF	Patient	Site*	Optical properties		
		$\mu_{a}$ (${\mathrm{cm}}^{-1}$)	$\mu_{s}^{\prime }$ (${\mathrm{cm}}^{-1}$)				$\mu_{a}$ (${\mathrm{cm}}^{-1}$)	$\mu_{s}^{\prime }$ (${\mathrm{cm}}^{-1}$)	CF
#007	AS	0.24	11.6	0.80	#038	PM	0.23	6.8	1.06
	PS	0.37	16.6	0.81		PS	0.32	12.3	0.89
#008	Apex	0.32	7.7	1.19		Apex	0.38	8.4	1.24
	PCW	0.16	9.1	0.75		ACW	0.19	12.5	0.69
#012	Apex	0.12	14.7	0.55	#040	PM	0.25	10.2	0.87
	PM	0.24	13.1	0.75		AS	0.35	11.2	0.98
#014	PS	0.15	13.4	0.61		Diaph	0.41	12.5	1.00
	Peri	0.09	12.5	0.53		Peri	0.42	9.1	1.25
#016	Apex	0.08	7.1	0.63	#047	Peri	0.23	12.6	0.75
	PCW	0.09	11.9	0.54		PM	0.32	9.5	1.04
#017	Apex	0.42	9.0	1.26		PS	0.24	10.8	0.83
	PCW	0.26	8.8	0.97		AS	0.26	9.9	0.90
	PM	0.24	10.9	0.82	#049	Peri	0.52	7.9	1.58
	ACW	0.33	12.4	0.90		Apex	0.34	8.8	1.13
#018	PS	0.44	5.9	1.76		ACW	0.52	10.2	1.32
	Apex	0.44	5.8	1.79		PCW	0.34	9.9	1.04
	PCW	0.70	7.4	2.04	#050	ACW	0.27	9.4	0.95
	ACW	0.72	5.9	2.50		PM	0.32	10.9	0.95
#020	PS	0.57	9.0	1.53		Apex	0.47	9.3	1.32
	Apex	0.27	9.0	0.98		Peri	0.22	12.3	0.74
	PCW	0.32	10.9	0.95	#052	PM	0.25	13.06	0.76
	PM	0.42	10.3	1.15		AS	0.47	13.17	1.05
#027	ACW	0.32	8.9	1.08		Diaph	0.35	10.23	1.04
	PM	0.26	11.9	0.81		ACW	0.39	13.45	0.93
	Apex	0.23	9.5	0.87		Apex	0.36	11.22	1.00
	Peri	0.26	9.8	0.91		PCW	0.27	10.86	0.87
#029	ACW	0.38	9.6	1.14		Peri	0.34	12.17	0.92
	PCW	0.65	5.2	2.57		PS	0.29	13.18	0.81
	Diaph	0.14	13.2	0.60	#053	PM	0.38	10.89	1.05
	Apex	0.28	10.3	0.92		PCW	0.31	10.80	0.94
#032	Apex	0.21	6.43	1.04		ACW	0.27	10.69	0.88
	PCW	0.17	4.97	1.09		Apex	0.35	6.91	1.35
	PM	0.18	6.33	0.97		Peri	0.33	8.59	1.12
	ACW	0.18	4.95	1.13		PS	0.32	7.92	1.17
#035	AS	0.38	9.1	1.18		AS	0.22	9.15	0.87
	PM	0.48	9.2	1.35		Diaph	0.28	9.12	0.99
	Peri	0.25	10.2	0.87	mean ± SD		0.31 ± 0.13	9.9 ± 2.4	1.03 ± 0.36
	Diaph	0.22	8.1	0.93					
#037	PM	0.17	9.9	0.74					
	Apex	0.22	9.3	0.86					
	ACW	0.33	9.8	1.03					
	PCW	0.26	9.5	0.93					

* See Sec. 2.1 and Fig. 1 for detailed description of sites.

**Fig. 5:**
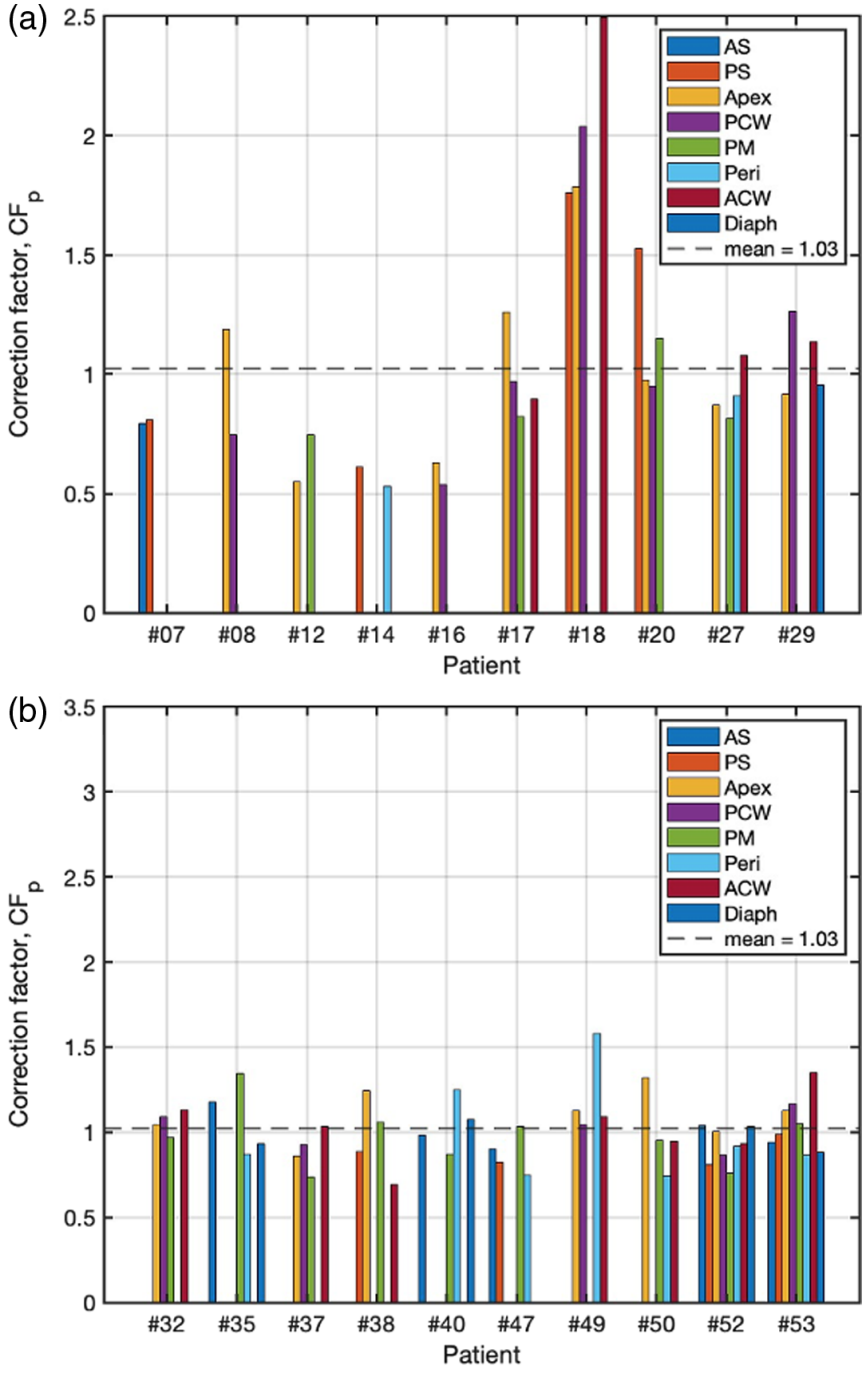
CF at 78 different sites in the pleural cavities of 20 patients. (a) Patient #07 to #29. (b) Patient #32 to #52. The mean value is represented by the dashed line.

The maximum variation of $\mu_{a}$ and $\mu_{s}^{\prime }$ is 8 times and 2.8 times, respectively, among all sites with a mean (SD) of $\mu_{a}=0.31$ (0.13) and $\mu_{s}^{\prime }=9.9$ (2.4) ${\mathrm{cm}}^{-1}$, respectively. The variations in tissue optical properties can be attributed to factors such as thermal damage to the surrounding tissue induced by electrosurgery during tumor resection and the diverse characteristics of cellular content and chromophores throughout the tissues.^38^ These factors are uncontrollable during the treatments, necessitating post-treatment correction. To provide a comprehensive understanding and address the influence of optical properties, correction factors derived from measurements taken at 78 sites across 20 patients at the treatment wavelength are illustrated in Fig. 5. This includes data from earlier cases using 2- or 4- channel PDT dose dosimetry systems. CFs were computed for individual sites using Eq. (1) to adjust the measured fluorescence for distortions arising from variable optical properties at different sites and among different patients. This correction is essential for ensuring the accurate quantification of PS concentration. The CFs range from 0.54 to 2.57 across the 78 sites. The mean CF value is 1.0 (indicated by the dashed line), and the median CF value is 1.08. The values for each site are presented in Table 1. The maximum variation among all sites is 4.7 times, and the maximum variation among sites within individual patients is 1.8 times.

### 3.2 Temporal and Spatial Distribution of Photofrin and PDT Dose

**Fig. 6:**
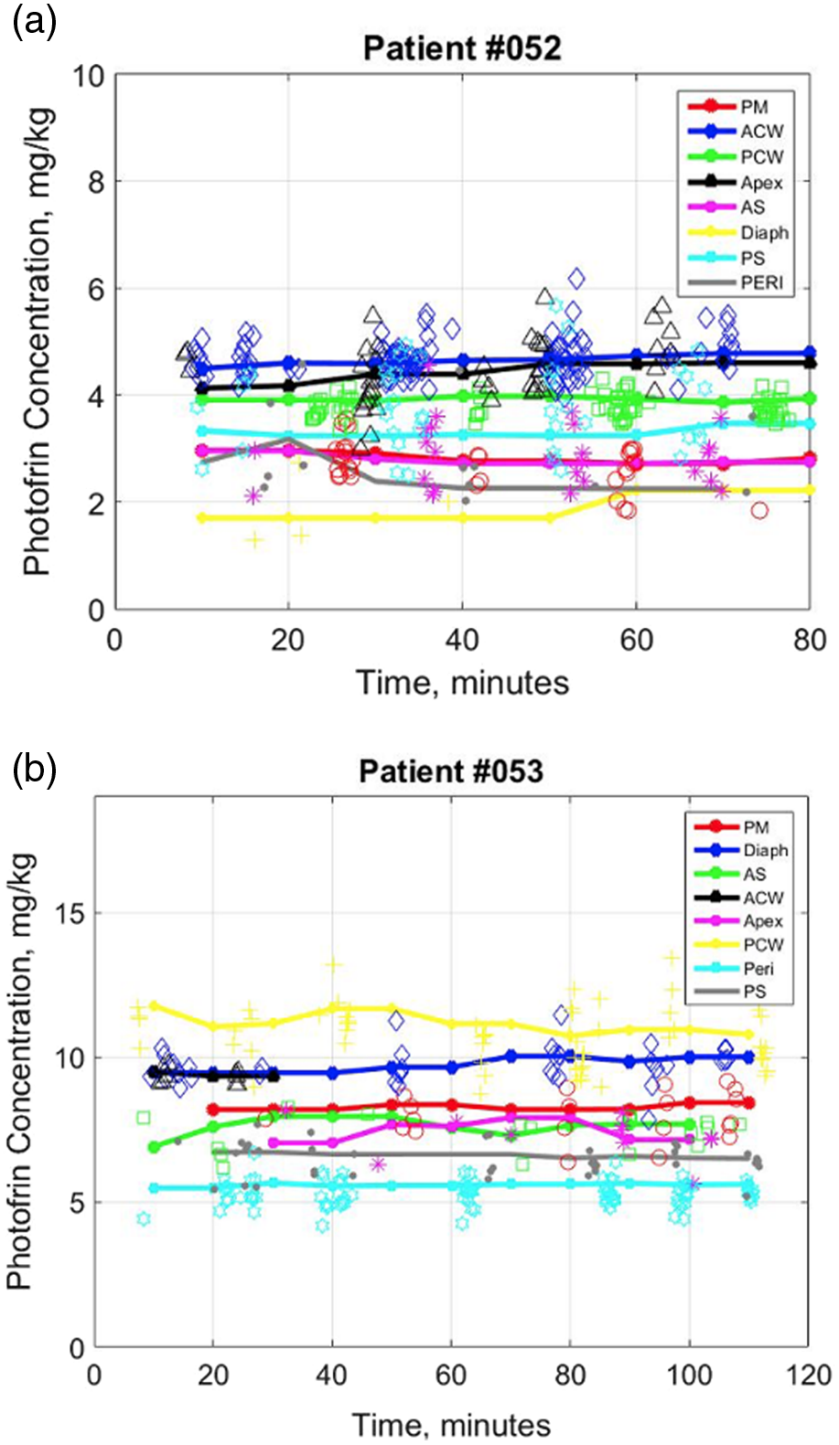
Temporal changes of Photofrin concentrations measured from 16 sites in the pleural cavity of the 2 most recent patients (#052 and #053) during the PDT treatments. To convert from ${\mathrm{mg}\;\mathrm{kg}}^{-1}$ to μM, $1\;{\mathrm{mg}\;\mathrm{kg}}^{-1}=1.65\;\mu M$ can be used.

Figure 6 shows the temporal changes in local Photofrin concentrations for 16 sites in the most recent two cases, in which all eight dual-function channels were utilized. The discrete data points in the graph were obtained from measured fluorescence spectra using the SVD fitting method described earlier. It is worth noting that some data points exhibit larger variations, which can be attributed to the fluctuations in the incident treatment light fluence rate (ranging from 0 to $1200\;{\mathrm{mW}/\mathrm{cm}}^{2}$). This variation stems from the movement of the light source during PDT, which introduces errors in the measurement of Photofrin fluorescence excited by the treatment light.

To facilitate a clearer observation of the trend of Photofrin variation during the treatment, the fitted data points for each channel were smoothed by calculating the average of all data points every 10 min of treatment time. The smoothed results are depicted by the solid curves in the graph. The relatively small temporal fluctuations in Photofrin concentration throughout the treatment for each site suggest that, despite the significant variations observed between different locations, the changes in Photofrin uptake over time within each site remain relatively stable. This phenomenon suggests that the uptake of Photofrin is not significantly influenced by the treatment itself but exhibits variation across different locations. The observed differences in optical properties ($\mu_{a}$ and $\mu_{s}^{\prime }$, tabulated in Table 1 for each patient) at these locations can be attributed to the inherent complexity of the tissue environment. This observation aligns with the substantial variation in CFs (see Fig. 5), calculated based on the tissue optical properties at the sites, as described in Sec. 3.1. Importantly, there is no significant photobleaching of Photofrin during PDT, corroborating findings from previous studies.^26^

**Fig. 7:**
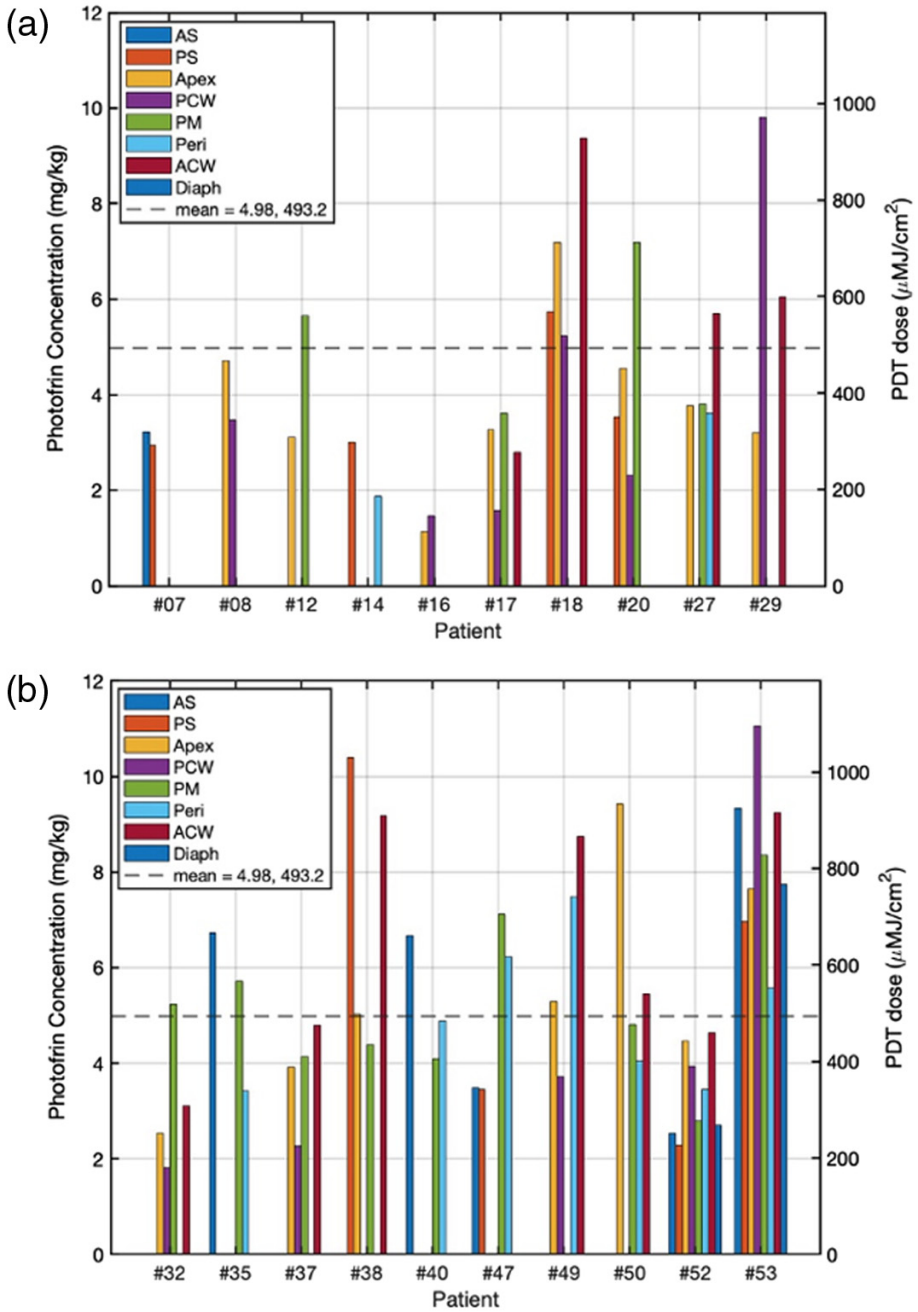
Mean Photofrin concentrations and PDT dose delivered to 78 different sites in the pleural cavities of 20 patients. (a) Patient #07 to #29. (b) Patient #32 to #52. The mean value is represented by the dashed line.

The mean Photofrin concentrations (shown on the left axis) and PDT doses (shown on the right axis) obtained from all 78 sites in the pleural cavities of 20 patients are shown in Fig. 7. The average (SD) and median Photofrin concentrations for all 78 sites are 4.98 (2.38) μM and 4.47  μM, respectively. The PDT dose delivered to each site is defined as the product of the local Photofrin concentration ([PS], in μM) and the delivered light dose. The treatment for each site was halted when the measured total light fluence of $60\;J/{\mathrm{cm}}^{2}$ was reached, ensuring a consistent total fluence across all subjects:

$$\mathrm{PDT}\text{ dose }(\frac{\mu \mathrm{MJ}}{{\mathrm{cm}}^{2}})=[\mathrm{PS}](\mu M)\cdot 60\;(\frac{J}{{\mathrm{cm}}^{2}}).$$ (5)

The differences in effective PDT doses delivered to different sites were influenced by the heterogeneities in Photofrin uptakes along the pleural cavities. The delivered PDT doses for each site are shown on the same plot (Fig. 6) with a secondary vertical axis on the right to indicate the corresponding values. The average (SD) and median PDT doses for all 78 sites are 493 (236) $\mu \mathrm{MJ}/{\mathrm{cm}}^{2}$ and $442.79\;\mu \mathrm{MJ}/{\mathrm{cm}}^{2}$, respectively. The maximum variation among all sites is 9.8 times, indicating significant differences in the delivered PDT doses across the pleural cavities. Similarly, the maximum variation among sites within each patient is 3.1 times, highlighting the variability in PDT doses within individual patients.

The substantial variations observed within and among patients align with previous studies and underscore the importance of considering both Photofrin concentration and PDT dose in the development of an effective dosimetry system. Traditional approaches that solely rely on monitoring light fluence may not provide comprehensive information to ensure uniform PDT doses across all treatment sites. One potential limitation in real-time monitoring of Photofrin concentration is the time-consuming post-treatment fitting and analysis of measured fluorescence spectra. However, this study demonstrates relatively stable Photofrin concentrations throughout the time course of PDT, enabling the utilization of data acquired at the beginning of treatment for comprehensive real-time guidance throughout the rest of the procedure. Therefore, the innovative 8-channel PDT dose dosimeter holds the potential to assist physicians in delivering more uniform PDT doses to all sites, ultimately improving the outcomes of pleural PDT treatment.

### 3.3 Real-Time Navigation System

**Fig. 8:**
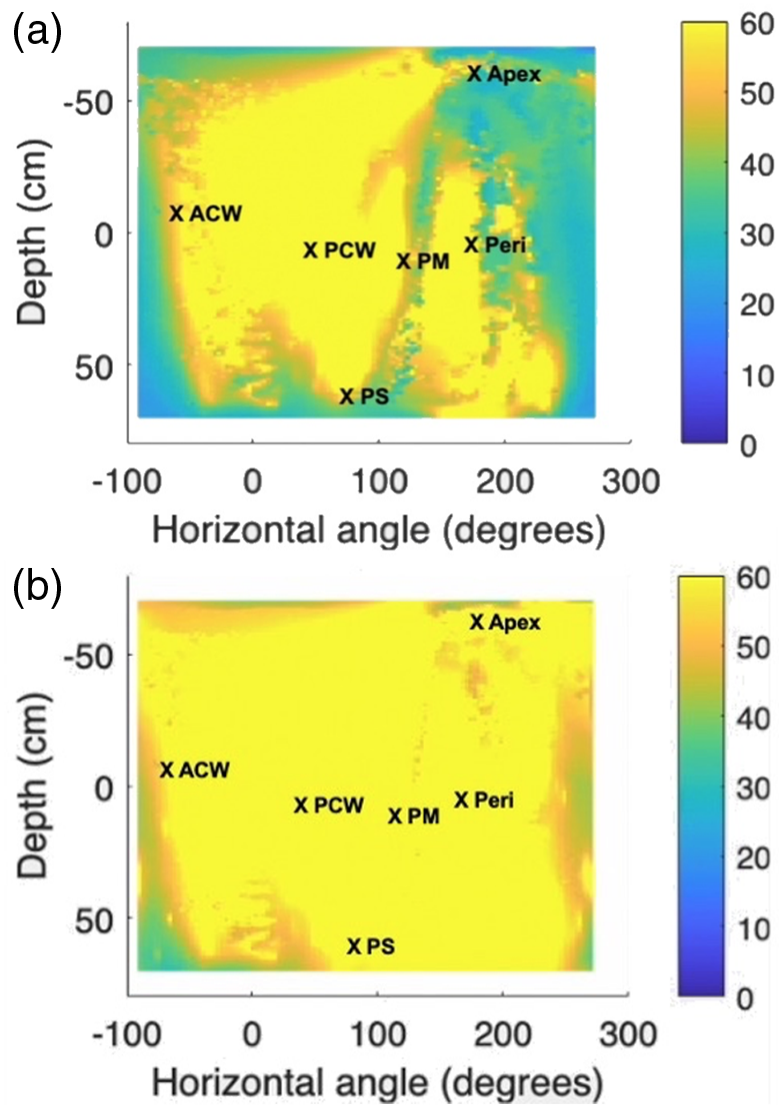
Comparison of light fluence map at the end of treatment with six detector positions labeled. (a) Without real-time fluence map guidance. (b) With real-time fluence map guidance. The overall light fluence distribution presented for the whole surface area was calculated based on the navigation system and Eqs. (3) and (4). Light fluence data were measured by the isotropic detectors at the locations marked with “X” to verify the calculations.

The preliminary results for light delivery efficiency with and without real-time guidance using the scanning and navigation system are depicted in Fig. 8. A pleural phantom (see Fig. 1), created using a 3D printing technique and based on a CT scan of an actual human pleural cavity, was utilized in our real-time navigation study. To simulate clinical conditions more accurately, six isotropic detectors were placed at predetermined locations on the inner surface of the pleural phantom (Apex, PCW, ACW, PS, PM, Peri) and connected to the light dosimetry system. The positions “Diaph” and “AS” were not included due to the positioning of the opening on the phantom, which allowed the scanner and treatment light source access. By comparing the results, it can be observed that employing the scanning and navigation system leads to a more uniform overall light distribution compared with PDT treatment using detectors alone. Although the prescribed light doses are accurately achieved at the crucial positions where the isotropic detectors are fixed, there are some remaining “cold spots” with lower light fluence. The innovative real-time navigation system effectively addresses this issue and enables the achievement of uniform light fluence distribution by the end of the treatment. The infrared (IR) navigation system has been utilized in previous clinical pleural PDT trials to track the motion of the light source during PDT and evaluate the uniformity of light fluence distribution across the entire pleural cavity after treatment. This serves as a baseline for the application of the updated navigation system, which incorporates rapid scanning and real-time feedback capabilities. The integration of these advancements can further enhance the accuracy and effectiveness of pleural PDT procedures.^39^

The real-time light fluence calculation of the novel system was validated by comparing the calculation with the data measured by all six detectors used in the phantom studies with different optical properties. To account for the general scattering inside the cavity and reduce the percentage error, a constant “b” was introduced based on previous studies. In this study, the scatter component was applied after treatment. The percentage error from the measured light dose at the end of treatment with real-time calculated light fluence using the primary component only [Eq. (1)] and the primary and scattering component together [Eq. (2)], along with the respective “b” values used, are summarized in Table 2. The variation in “b” values was found to be small among previous clinical cases, and this study confirms the same observation. After applying the scatter component, the range of the percentage error in light fluences is between 0.9% and 12.8%, which is comparable to the findings of previous studies (ranging from 0.7% to 15.4%).^23^ This study demonstrates the improvement in light delivery efficiency achieved by utilizing the real-time navigation system, and the algorithm employed is validated. The validation process affirms the accuracy and reliability of the real-time light fluence calculation, establishing its suitability for application in pleural PDT procedures. This establishes a foundation for the development of a comprehensive real-time guidance system, integrating the 8-channel dosimetry system with the navigation system. Through the combination of real-time calculated light fluence data for the pleural cavity and simultaneously measured PS concentrations at eight crucial sites, our objective is to furnish physicians with real-time PDT dose information. This information serves as a guidance tool for optimizing light delivery during PDT, with the overarching goal of further advancing treatment efficiency.

**Table 2:** Percent error from measured light dose at the end of treatment with the calculated light fluence using (a) the primary component only and (b) the primary plus scattering component. Here, the study 1, 2, and 3 refer to liquid phantoms with different optical properties, i.e., $\left(\mu_{a},\mu_{s}^{\prime })=\;(0.1,2\right)$, (0.5, 10), and (1, 20) ${\mathrm{cm}}^{-1}$.

Study		Peri (%)	PM (%)	ACW (%)	PCW (%)	PS (%)	Apex (%)	B (${\mathrm{mWcm}}^{-2}$)
(a)	1	33.7	33.5	32.8	15.2	26.7	14.2	NA
	2	22.7	33.1	35.0	24.7	15.3	24.9	NA
	3	31.6	31.5	21.9	15.6	28.9	17.4	NA
(b)	1	3.3	5.3	12.8	4.2	7.1	1.4	6.5
	2	7.1	3.0	5.3	0.9	4.8	3.1	6.8
	3	1.3	3.9	9.2	17.9	2.9	7.6	6.5

## 4 Conclusions

In this study, a real-time 8-channel PDT dose dosimeter was developed and utilized for Photofrin-mediated pleural PDT. The mean PDT dose among the 20 patients included in the study was found to be $493.17\;\mu \mathrm{MJ}/{\mathrm{cm}}^{2}$. However, it was observed that, with the same administered Photofrin dose and total light dose, PDT doses could vary significantly, ranging up to 980% among different patients and 310% within the same patient. This variability implies that, relying solely on light fluence as the treatment guidance during PDT leads to a non-uniform distribution of PDT dose throughout the pleural cavity, potentially impacting the overall treatment outcome. Recognizing PDT dose as a crucial dosimetry parameter, it can serve as a superior guiding tool for physicians during PDT. To address this issue and improve the consistency of PDT doses, the 8-channel dose dosimeter was developed, providing a means to initiate PDT dose-mediated pleural PDT. The system incorporated commercial tracking and scanning tools along with customized software to enable real-time feedback and monitoring. The scanning and navigation system efficiently identified the target surface and facilitated quick registration for real-time light fluence distribution calculation during PDT. The developed system has been successfully applied in phantom studies, demonstrating its ability to provide reliable real-time feedback on the light source position and 2D light fluence distribution.

The significance of the 8-channel system lies in its potential to provide informative real-time guidance at all sites currently used in the clinical trial. By addressing variations in PDT doses and ensuring uniform dose distribution, the system offers a promising avenue for starting a new clinical trial utilizing PDT dose as dosimetry metrics. Future development will prioritize the integration of the 8-channel dosimetry system with the scanning and navigation system to guide light delivery during PDT. This integration aims to guarantee the consistent distribution of PDT dose, thereby further elevating the effectiveness of cancer treatment outcomes.

## Data Availability

Data underlying the results presented in this paper may be obtained from the authors upon reasonable request.
